# Monitoring of Carbon Dioxide Using Hollow-Core Photonic Crystal Fiber Mach–Zehnder Interferometer

**DOI:** 10.3390/s19153357

**Published:** 2019-07-31

**Authors:** Farid Ahmed, Vahid Ahsani, Kaveh Nazeri, Ehsan Marzband, Colin Bradley, Ehsan Toyserkani, Martin B. G. Jun

**Affiliations:** 1 Multi-Scale Additive Manufacturing Laboratory, Department of Mechanical and Mechatronics Engineering, University of Waterloo, Waterloo, ON N2L3G1, Canada; 2 Department of Mechanical Engineering, University of Victoria, Victoria, BC V8W2Y2, Canada; 3 School of Mechanical Engineering, Purdue University, West Lafayette, IN 47907, USA

**Keywords:** carbon dioxide gas, fiber-optic sensor, Mach–Zehnder interferometer, photonic crystal fiber

## Abstract

Monitoring of greenhouse gases is essential to understand the present state and predict the future behavior of greenhouse gas emissions. Carbon dioxide (CO_2_) is the greenhouse gas of most immediate concern, because of its high atmospheric concentration and long lifetime. A fiber-optic Mach–Zehnder interferometer (MZI) is proposed and demonstrated for the laboratory-scale monitoring of carbon dioxide concentration. The interferometric sensor was constructed using a small stub of hollow-core photonic crystal fiber between a lead-in and lead-out standard single mode fiber, with air-gaps at both interfaces. At room temperature and atmospheric pressure, the sensor shows the sensitivity of 4.3 pm/% CO_2_. The device was packaged to demonstrate the laboratory-scale leakage detection and measurement of CO_2_ concentration in both subsurface and aqueous environments. The experimental study of this work reveals the great potential of the fiber-optic approach for environmental monitoring of CO_2_.

## 1. Introduction

Carbon dioxide (CO_2_) has been identified as the primary heat-trapping gas to adversely affect our climate between 1750 and 2011 [1]. As our planet is likely to face greater future challenges, in recent years considerable carbon mitigation research has been undertaken in an effort to fight global warming caused by CO_2_. Deployment of carbon capture and storage (CCS) technologies have been proposed for a drastic reduction of CO_2_ emission [2]. As of September 2012, 75 large-scale global CCS projects (at least 400,000 tons of CO_2_ per year) and a number of projects under advanced stages of development have been identified by the Global CCS Institute [3]. While CCS has a vital role in controlling greenhouse gas emissions, CO_2_ leakage at sequestration sites is the primary concern because of its adverse environmental impacts [4,5]. Research models predict that in an event of leakage, CO_2_ escape rate above 0.1% may jeopardize the effectiveness of a geological storage site [6]. Hence, early detection of CO_2_ leakage is essential to re-establish the efficiency of a CCS operation and minimize its ecological damages. Monitoring of CO_2_ leakage has been investigated using diverse approaches including seismic [7], geoelectrical [8], geochemical [9], gravimetric [10], temperature logs [11], and soil gas composition [12]. There are many challenges inherent to monitoring a CCS project, most notably: prolonged periods (i.e., several decades), long transmission lengths, extreme physical and chemical conditions, and capital and operating costs. Most of the techniques mentioned above have a low sensing resolution or do not meet the challenges associated with monitoring CCS projects.

Fiber optic sensors have unique properties that make them ideal for CCS site monitoring, for example: compact size; immunity to electromagnetic radiation; superior aging characteristics; long-haul monitoring capabilities; and, the ability for high-resolution CO_2_ detection. Fiber optic sensors are very efficient in gas detection and monitoring applications [13,14,15] as compared to conventional sensors. Various fiber-optic sensor configurations have been exploited for monitoring CO_2_ concentration, including long period grating [16], Bragg grating [17], evanescent filed [18], and fluorescent based [19,20]. Fiber fluorescence sensor has also been employed to demonstrate monitoring of environmental CO_2_ [21]. Use of fluorescent dyes, nanoparticle coatings, or extensive etching of the fibers in the reported studies are likely to affect the aging behavior and robustness of the sensors. There has been a constant effort to develop highly sensitive all-fiber sensors using suitable fiber type and device configuration [13,14,22]. The evanescent field in the cladding air holes of photonic crystal fibers reveals new opportunities to exploit light interaction with gasses for sensing applications [23,24]. Hollow-core photonic crystal fibers (HC-PCFs) have air holes running along the fiber propagation axis in both the core and cladding regions. These structures have been introduced to improve the light interaction with the sample gas leading to superior sensitivity [25,26]. Various mechanisms have been explored for HC-PCF-based gas sensing, including tunable modal interference [27], Fabry–Pérot interference [28], photothermal phase modulation [29], surface-enhanced Raman scattering [30], and a gas absorption spectroscopy [25,31]. However, these sensing devices require an extended length of HC-PCF, drilling side micro-holes on HC-PCF, or filling the air holes of HC-PCF with a Raman scattering substrate such as nanoparticle colloids. The reported fiber-optic gas sensors to date either display insufficient sensitivity/reliability or require complex fabrication and/or packaging processes.

**Figure 1:**
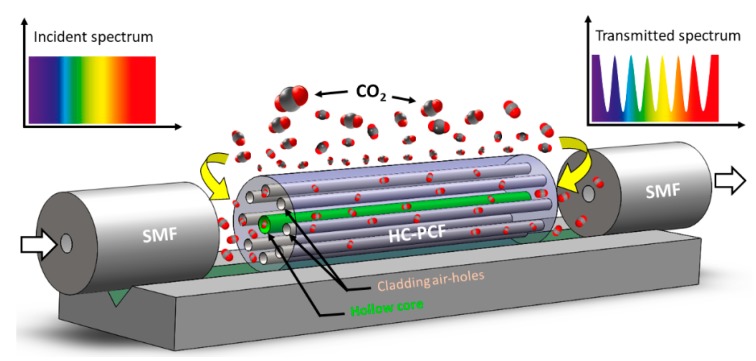
Schematic of the Mach–Zehnder interferometer (MZI) constructed using a small stub of hollow-core photonic crystal fiber (HC-PCF).

In this study, a HC-PCF-based Mach–Zehnder interferometer (MZI) is proposed for the detection and measurement of CO_2_ concentration in subsurface soil and aqueous environments. As shown in Figure 1, the device was fabricated by placing a small stub of HC-PCF between a lead-in and lead-out standard single mode fiber (SMF) with air gaps at both interfaces. The air holes in HC-PCF provides a large area of interaction with the gas leading to high-sensitive detection and measurement of CO_2_.

## 2. Working Principle

**Figure 2:**
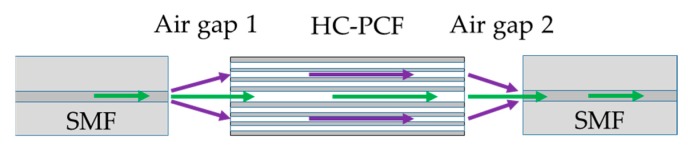
Working principle based on geometric light propagation in the fiber assembly.

The schematic of the sensor configuration is shown in Figure 2. It is a common MZI structure where the fringe pattern appears at the transmission end because of interference between the core mode and dominant cladding mode. The transmission fringe of a fiber-optic MZI can be expressed as [32,33]:

(1) $$I=I_{core}+I_{clad}+2\sqrt{I_{core}I_{clad}}\cos\phi ,$$

where $I_{core}\;$ and $I_{clad}$ are the intensities of light in the core and cladding, respectively. The phase difference between core and cladding mode can be expressed as $\phi =2\pi \Delta n_{eff}L/\lambda$, where $n_{eff}$ is the difference of effective refractive indices between core and cladding modes, *L* is the length of the MZI, and *λ* is the wavelength. For ϕ=(2m+1)π in Equation (1), the *m^th^* order attenuation peak is maximum and its associated wavelength can be expressed as $\lambda_{m}=2\Delta n_{eff}L/\left(2m+1\right)$. Therefore, any change in $\Delta n_{eff}$ or *L* due to an ambient refractive index or temperature variation results in a shift of attenuation peak. Such spectral shifts can be utilized to measure the ambient gas concentration.

## 3. Sensor Fabrication and Packaging

**Figure 3:**
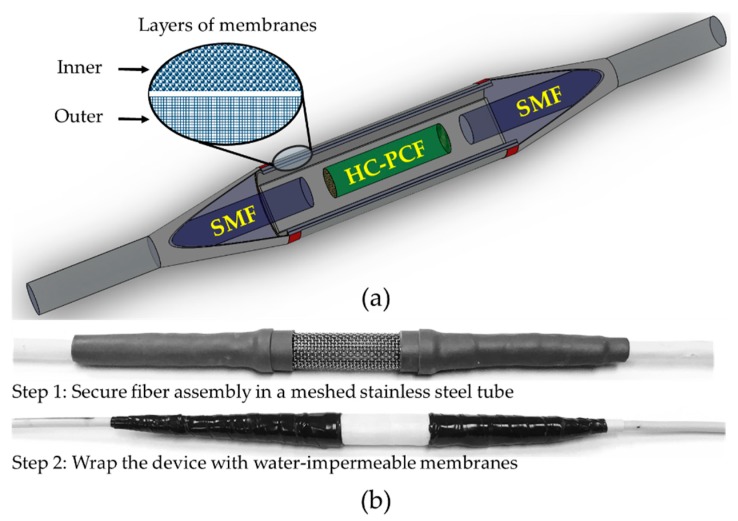
(**a**) Schematic of packaging, and (**b**) sensor packaging.

**Figure 4:**
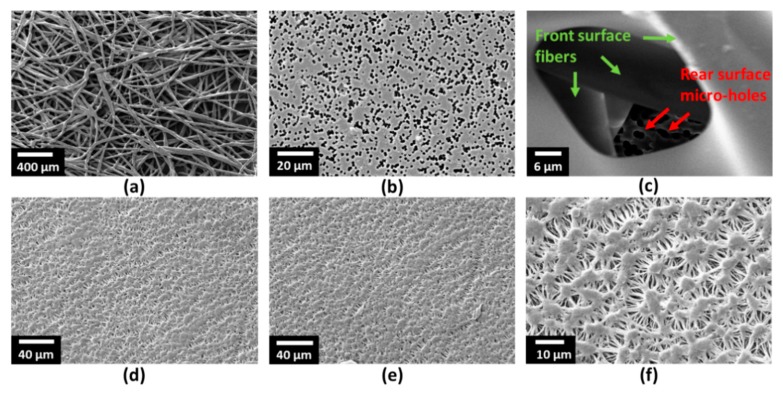
SEM images of the two membranes used in packaging of the sensor to make it waterproof: (**a**) Front surface of the inner membrane, (**b**) rear surface of the inner membrane, (**c**) magnified image of the inner membrane showing both fibers and micro-holes on two surfaces, (**d**) front surface of the outer membrane, (**e**) rear surface of the outer membrane, and (**f**) the magnified image if the outer membrane.

As shown in Figure 1, SMF (SMF-28, ThorLabs, Newton, NJ, USA) and HC-PCF (HC-1500, NKT Photonics, Birkerød, Denmark) were used to fabricate the sensor. The core diameter, cladding pitch, diameter of PCF region, and cladding diameter of the HC-PCF were 10 ± 1 µm, 3.8 ± 0.1 µm, 70 ± 5 µm, and 120 ± 2 µm, respectively. The design wavelength and numerical aperture of the HC-PCF were 1550 nm and ~0.2, respectively. The small stub of HC-PCF (4.30 mm) was placed between a lead-in and lead-out SMFs with suitable air gaps (air gap 1:2.24 mm and air gap 2:1.83 mm in Figure 2) at both interfaces. The fiber assembly was first aligned and fixed on V-groove of a glass slide (25 × 5 × 1 mm) using a waterproof adhesive. Suitable packaging of a fiber-optic sensor is crucial for its reliable sensing operation and longer life span. Therefore, the device was packaged to make it operational in the subsurface and aqueous environment. Figure 3a,b show the schematic of the packaged configuration, and the steps involved in packaging the sensor, respectively. The glass slide holding the fiber assembly was first secured in a stainless steel mesh tube to provide mechanical strength to the fiber assembly and allow easy handling of the device. The sensor was then wrapped with two water-impermeable membranes that allow gas flow in the chamber. The scanning electron microscope (SEM) images shown in Figure 4 reveal the morphologies of both front and rear surfaces of the inner (TRAKETCH, Sabeu GmbH & Co. KG, Northeim, Germany) and outer (SQ-S GASKET SHEET, Inertech Inc., Monterey Park, CA, USA) membranes. Figure 4a–c shows the front, rear, and magnified images of TRAKETCH membrane capturing both surfaces, respectively. The capillary pores (pore size: 1.37 ± 0.02 µm, pore density: ~13.7 × 10^6^/cm^2^, thickness: 170 ± 20 µm, water entry pressure ≥0.6 bar) of this micro filtration membrane work as hydrophobic surface and allow CO_2_ gas flow. Figure 4d–f shows the front, rear, and magnified surfaces of the INERTEX SQ-S membrane. The gasket sheet is made of polytetrafluoroethylene (PTFE) that can operate in full vacuum to 200 bar pressure, and it does not support any bacterial growth. The two membranes were assembled to achieve a good balance of CO_2_ permeability and water resistance. To protect the device from dirt and dripping water, it was finally wrapped with Tyvek, a commercially-available building water-insulation membrane.

## 4. Results and Discussion

### 4.1. Sensor Characterization

**Figure 5:**
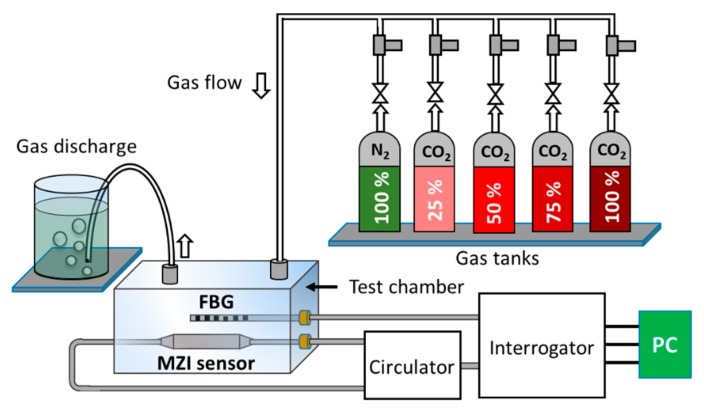
Experimental schematic for sensor characterization and interrogation.

**Figure 6:**
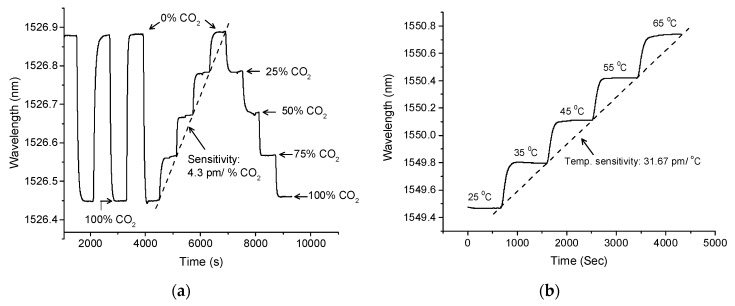
The response of the sensor to (**a**) CO_2_ concentration, and (**b**) temperature change.

The schematic shown in Figure 5 was used to characterize CO_2_ concentration in near atmospheric pressure. The dimension of the test chamber used in this experiment was 14.5 × 11.2 × 4.4 cm. The sensor was interrogated with different known concentrations (25%, 50%, 75%, and 100%) of CO_2_ gases. A 99.99% pure nitrogen (N_2_) was used as a reference gas and the discharge tube with a bubbler was used to keep near atmospheric pressure in the test chamber. A circulator was used to connect the MZI sensor to the micron optics interrogator (SM125). The chamber was also equipped with a fiber Bragg grating (FBG) to monitor any temperature variation during the experiments. As shown in Figure 6a, the sensor shows a consistent trend of shifts as it is subjected to different concentration of CO_2_ gas. As calculated from the slope of Figure 6a, the sensor has a sensitivity of 4.33 pm/% CO_2_. Considering the sensitivity of the sensor and wavelength stability of the interrogator (1 pm), it exhibits a measurement resolution of ~0.2% CO_2_, which is 10 times better compared to a recently-reported all-optical sensor constructed using HC-PCF [34]. Figure 6b shows the sensor response to the ambient temperature change in the range of 25 to 65 °C. The sensor shows a linear spectral shift with temperature change with a sensitivity of 31.67 pm/°C.

**Figure 7:**
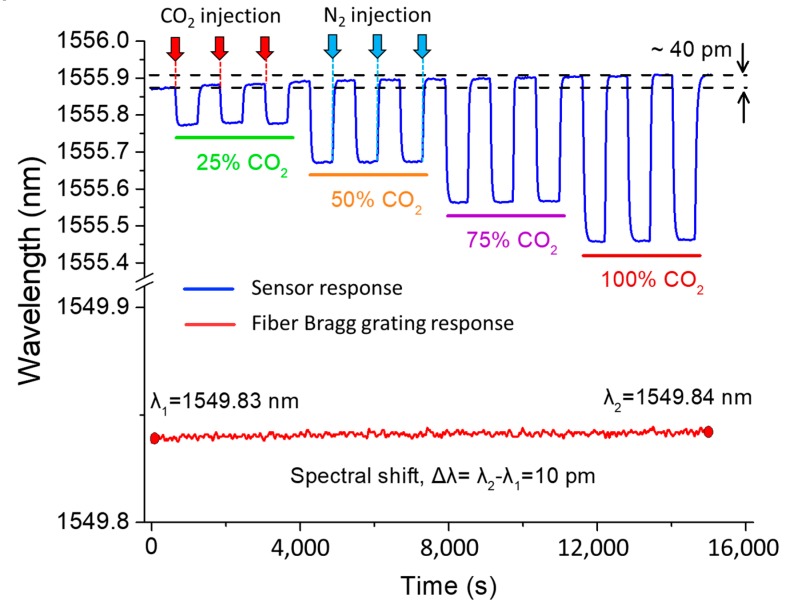
Cyclic test to examine the measurement reliability of the gas sensor.

Reliable operation is essential in any sensing application. Hence, repeatable sensing capability is crucial in particular for effective gas monitoring applications. To examine the sensing reliability of the device, a cyclic test was performed for four different known concentration of CO_2_ gasses such as 25% CO_2_, 50% CO_2_, 75% CO_2_, and 100% CO_2_, as shown in Figure 7. Nitrogen was used as a reference gas for this test. The cyclic test started with injecting atmospheric N_2_ gas into the test chamber for 10 min. Once the injection of N_2_ stopped, the 25% CO_2_ gas was injected into the chamber for 10 min. This two-step process was repeated two more times to finish the cyclic test for 25% CO_2_. For the higher concentration of CO_2_ gases, the same two-step process was repeated three times. An FBG sensor with a temperature sensitivity of 10 pm/°C was placed in the test chamber to monitor any temperature change during this experiment. The sensor shows good repeatability in the measurement of CO_2_ concentration. A maximum of 40 pm spectral deviation was observed for the entire experiment as shown in Figure 7, which is largely because of temperature change in the chamber. The FBG shows 1 °C increase of temperature (that corresponds to 10 pm FBG spectral shift) in the test chamber by the end of the experiment. Since the temperature sensitivity of the proposed MZI sensor (31.67 pm/°C) is about three times more than that of FBG (10 pm/°C), ~32 pm spectral shift appeared due to 1 °C temperature raise in the test chamber. The remaining 8 pm spectral shift may appear due to a pressure change in the chamber or measurement errors. The sensor showed good measurement repeatability, as shown in Figure 7.

**Figure 8:**
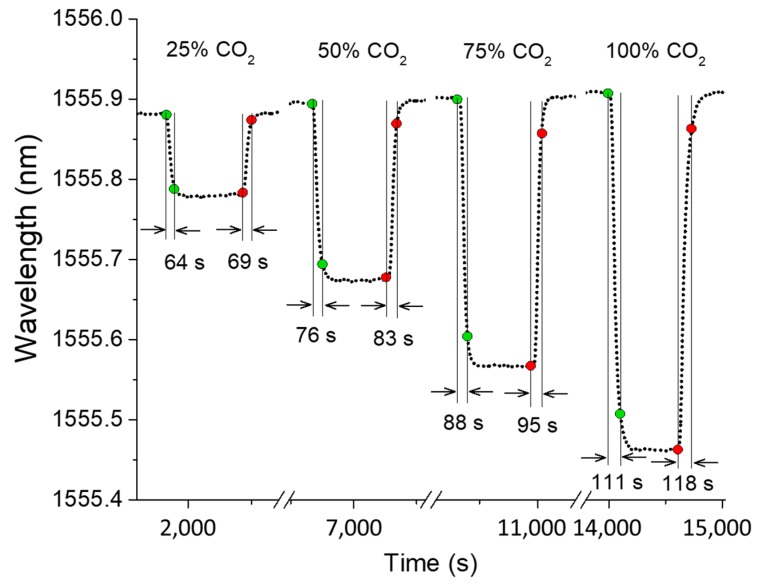
Response and recovery times of the sensor for different concentration of CO_2_ gases.

Response and recovery times are widely used to characterize gas sensors, which are essentially the times taken by a sensor to reach 90% of final and initial indications, respectively. For the given test chamber, Figure 8 reports the response and recover times for four different concentration of CO_2_ gases. The response times of the sensor for 25%, 50%, 75%, and 100% CO_2_ are 64, 76, 88, and 111 s, respectively. The recovery times of the sensor for 25%, 50%, 75%, and 100% CO_2_ are 69, 83, 95, and 118 s, respectively. Since the sensor sits on the floor of the test chamber and the CO_2_ gases used in this experiment are heavier than the reference gas (N_2_), response times are shorter than the recovery times. The main limiting factor for both response and recovery times is the delivery of gasses to the air gaps and air holes. Therefore, both the response and recovery times can be improved by reducing the volume of the test chamber.

### 4.2. Subsurface CO_2_ Measurement

**Figure 9:**
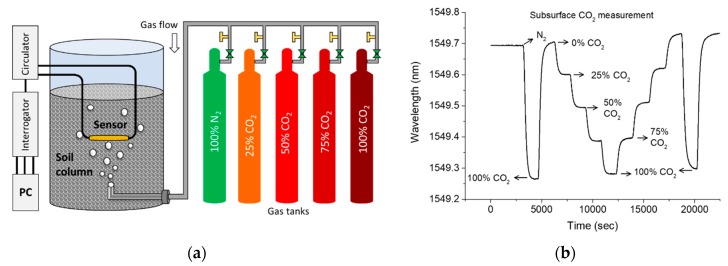
(**a**) Laboratory setup for CO_2_ concentration measurement in soil, and (**b**) measurement of CO_2_ concentrations in soil (at atmospheric pressure and room temperature).

The sensor was tested to measure CO_2_ concentration in soil column in room temperature and atmospheric pressure. As shown in the schematic of Figure 9a, the sensor was buried 20 cm under the 50 cm-deep soil column. The radius and height of the cylindrical soil column are 30 and 60 cm, respectively. The CO_2_ and N_2_ were injected into the soil column from the bottom. The transmission spectrum of the interferometer was first monitored for 10 min in the air, followed by injection of N_2_ for next 10 min, as shown in Figure 9b. The 100% CO_2_ was then injected into the soil column for 10 min followed by injecting reference N_2_ gas for another 10 min. The percentage of CO_2_ injection was then gradually increased up to 100% as shown in Figure 9b. A reverse trend was then followed to return the sensor spectrum to the initial level. The 100% CO_2_ gas was then injected for 10 min for the last time followed by injection of the reference gas. The purpose of this test was to understand the response of the sensor in the sub-surface environment. With a larger surface area of the soil column exposed to the atmosphere and increased volume of sensing environment, the sensor shows longer response and recovery times (with reference to Figure 6a). At the end of the experiment, the overall spectrum also followed a red shift, which may be attributed to the rise in temperature as also noticed in Figure 7.

### 4.3. Aqueous CO_2_ Measurement

**Figure 10:**
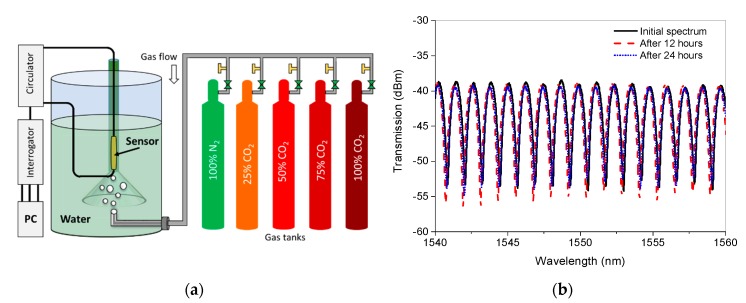
(**a**) Setup for CO_2_ concentration measurement in an aqueous environment, and (**b**) spectral stability in water for 24 h.

Figure 10a shows the experimental setup for the measurement of CO_2_ concentration in an aqueous environment. To investigate the performance of water-resistant membranes used packaging, the sensor was first immersed in water for 24 h and its transmission spectrum was saved for every 12 h. As shown in Figure 10b, the spectrum of the sensor did not show any significant degradation over the period of 24 h, except small spectral shift which is likely due to temperature change during the experiment.

**Figure 11:**
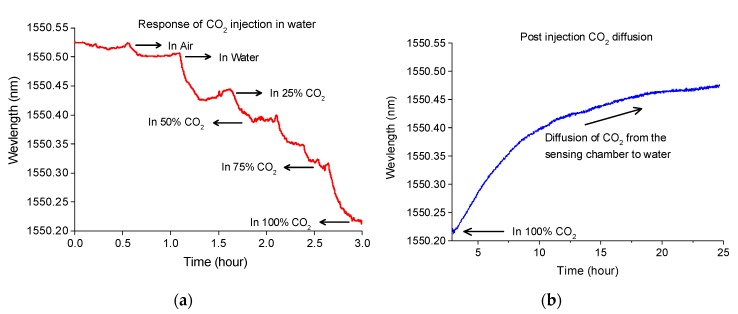
(**a**) The response of the sensor to different concentrations of CO_2_ in water, and (**b**) tracking slow diffusion of CO_2_ gas when injection stopped.

A funnel was used for guiding the CO_2_ bubbles to the sensor vicinity that helps the diffusion of gas into the packaged sensor. The response of the sensor to CO_2_ concentration changes in the aqueous environment is shown in Figure 11a. Each of the 25%, 50%, 75%, and 100% CO_2_ gases were successively injected for 30 min. The sensor shows spectral blue shifts with a gradual increase in CO_2_ concentration. Unlike the experiments in test chamber or soil column, the sensor showed irregular spectral shifts, which may appear due to the turbulence created by the CO_2_ bubbles. Once immersed in the water, the ambient humidity was considered stable during the experiment. Detailed effects of humidity in the aqueous environment will be conducted in the future, which may also explain the anomalies in the spectral shift during the experiment. After injecting 100% CO_2_ for 30 min, the injection was stopped to let CO_2_ gradually diffuse out of the packaged sensor. As shown in Figure 11b, diffusion of CO_2_ out of the packaged sensor is very slow. An injection of reference gas to the sensing chamber may significantly improve the recovery time for such monitoring applications.

## 5. Conclusions

In this study, an HC-PCF-based MZI is introduced that can measure ambient CO_2_ concentration for environmental monitoring applications. The device was constructed by placing a stub of HC-PCF between a lead-in and lead-out SMF. The sensor was packaged and characterized for known concentrations of CO_2_ gases. At room temperature and atmospheric pressure, the sensor shows a linear response to CO_2_ concentration with the sensitivity of 4.3 pm/% CO_2_. Considering the measurement device used in this study had a wavelength stability of 1 pm, the resolution of the sensor is ~0.2% CO_2_. For the test chamber dimension of 14.5 × 11.2 × 4.4 cm, the sensor shows fast response and recovery times of 64 and 69 s, respectively. Water-resistant but gas-permeable membranes were used to package the sensor. The sensing device demonstrated the laboratory-scale leakage detection and measurement of CO_2_ concentration in both subsurface and aqueous environments. The sensor showed a stable and reliable measurement of CO_2_ concentrations with a considerably short response and recovery times. The experimental study of this work reveals the potential of the fiber-optic approach for environmental monitoring of CO_2_ leakage and concentration.

## References

[1] Ciais P., Sabine C., Bala G., Bopp L., Brovkin V., Canadell J., Chhabra A., DeFries R., Galloway J., Heimann M. (2013). The physical science basis. Contribution of working group I to the fifth assessment report of the intergovernmental panel on climate change. Chang. IPCC Clim..

[2] Cuéllar-Franca R.M., Azapagic A. (2015). Carbon capture, storage and utilisation technologies: A critical analysis and comparison of their life cycle environmental impacts. J. CO_2_ Util..

[3] GCCSI (2012). The Global Status of CCS: 2012.

[4] Smith K.L., Steven M.D., Jones D.G., West J.M., Coombs P., Green K.A., Barlow T.S., Breward N., Gwosdz S., Krüger M. (2013). Environmental impacts of CO_2_ leakage: Recent results from the ASGARD facility, UK. Energy Procedia.

[5] Zhao X., Deng H., Wang W., Han F., Li C., Zhang H., Dai Z. (2017). Impact of naturally leaking carbon dioxide on soil properties and ecosystems in the Qinghai-Tibet plateau. Sci. Rep..

[6] Leung D.Y.C., Caramanna G., Maroto-Valer M.M. (2014). An overview of current status of carbon dioxide capture and storage technologies. Renew. Sustain. Energy Rev..

[7] Arts R., Eiken O., Chadwick A., Zweigel P., van der Meer L., Zinszner B. (2004). Monitoring of CO_2_ injected at Sleipner using time-lapse seismic data. Energy.

[8] Kiessling D., Schmidt-Hattenberger C., Schuett H., Schilling F., Krueger K., Schoebel B., Danckwardt E., Kummerow J. (2010). Geoelectrical methods for monitoring geological CO_2_ storage: First results from cross-hole and surface-downhole measurements from the CO_2_SINK test site at Ketzin (Germany). Int. J. Greenh. Gas Control.

[9] Riding J.B., Rochelle C.A. (2005). The IEA Weyburn CO_2_ Monitoring and Storage Project-Final Report of the European Research Team.

[10] Gerstenberger M., Nicol A., Stenhouse M., Allinson G., Berryman K., Doody B., Ho M., McCurdy M., Neal P., Stirling M. (2009). Opportunities for Underground Geological Storage of CO_2_ in New Zealand-Report CCS-08/10- Risk Assessment Methodologies.

[11] Bielinski A., Kopp A., Schütt H., Class H. (2008). Monitoring of CO_2_ plumes during storage in geological formations using temperature signals: Numerical investigation. Int. J. Greenh. Gas Control.

[12] Jenkins C.R., Cook P.J., Ennis-King J., Undershultz J., Boreham C., Dance T., de Caritat P., Etheridge D.M., Freifeld B.M., Hortle A. (2012). Safe storage and effective monitoring of CO_2_ in depleted gas fields. Proc. Natl. Acad. Sci. USA.

[13] Lee B. (2003). Review of the present status of optical fiber sensors. Opt. Fiber Technol..

[14] Udd E. (1995). An overview of fiber-optic sensors. Rev. Sci. Instrum..

[15] Kersey A.D. (1996). A review of recent developments in fiber optic sensor technology. Opt. Fiber Technol..

[16] Hromadka J., Tokay B., Correia R., Morgan S.P., Korposh S. (2018). Carbon dioxide measurements using long period grating optical fibre sensor coated with metal organic framework HKUST-1. Sens. Actuators B Chem..

[17] Shivananju B.N., Yamdagni S., Fazuldeen R., Sarin Kumar A.K., Hegde G.M., Varma M.M., Asokan S. (2013). CO_2_ sensing at room temperature using carbon nanotubes coated core fiber Bragg grating. Rev. Sci. Instrum..

[18] Chong X., Kim K.J., Ohodnicki P.R., Li E., Chang C.H., Wang A.X. (2015). Ultrashort Near-Infrared Fiber-Optic Sensors for Carbon Dioxide Detection. IEEE Sens. J..

[19] Wolfbeis O.S., Weis L.J., Ziegler W.E., Leiner M.J.P. (1988). Fiber-Optic Fluorosensor for Oxygen and Carbon Dioxide. Anal. Chem..

[20] Chu C.-S., Lo Y.-L., Sung T.-W. (2011). Review on recent developments of fluorescent oxygen and carbon dioxide optical fiber sensors. Photonic Sens..

[21] Wolfbeis O.S., Kovacs B., Goswami K., Klainer S.M. (1998). Fiber-optic fluorescence carbon dioxide sensor for environmental monitoring. Mikrochim. Acta.

[22] Lee B.H., Kim Y.H., Park K.S., Eom J.B., Kim M.J., Rho B.S., Choi H.Y. (2012). Interferometric fiber optic sensors. Sensors.

[23] Villatoro J., Kreuzer M.P., Jha R., Minkovich V.P., Finazzi V., Badenes G., Pruneri V. (2009). Photonic crystal fiber interferometer for chemical vapor detection with high sensitivity. Opt. Express.

[24] Hoo Y.L., Jin W., Shi C., Ho H.L., Wang D.N., Ruan S.C. (2003). Design and Modeling of a Photonic Crystal Fiber Gas Sensor. Appl. Opt..

[25] Ritari T., Tuominen J., Ludvigsen H., Petersen J.C., Sørensen T., Hansen T.P., Simonsen H.R. (2004). Gas sensing using air-guiding photonic bandgap fibers. Opt. Express.

[26] Hoo Y.L., Jin W., Ho H.L., Ju J., Wang D.N. (2005). Gas diffusion measurement using hollow-core photonic bandgap fiber. Sens. Actuators B Chem..

[27] Yang F., Jin W., Cao Y., Ho H.L., Wang Y. (2014). Towards high sensitivity gas detection with hollow-core photonic bandgap fibers. Opt. Express.

[28] Yang F., Tan Y., Jin W., Lin Y., Qi Y., Ho H.L. (2016). Hollow-core fiber Fabry–Perot photothermal gas sensor. Opt. Lett..

[29] Lin Y., Jin W., Yang F., Tan Y., Ho H.L. (2017). Performance optimization of hollow-core fiber photothermal gas sensors. Opt. Lett..

[30] Yang X., Shi C., Wheeler D., Newhouse R., Chen B., Zhang J.Z., Gu C. (2010). High-sensitivity molecular sensing using hollow-core photonic crystal fiber and surface-enhanced Raman scattering. J. Opt. Soc. Am. A Opt. Image Sci. Vis..

[31] Cubillas A.M., Silva-Lopez M., Lazaro J.M., Conde O.M., Petrovich M.N., Lopez-Higuera J.M. (2007). Methane detection at 1670-nm band using a hollow-core photonic bandgap fiber and a multiline algorithm. Opt. Express.

[32] Ahmed F., Ahsani V., Melo L., Wild P., Jun M.B.G. (2016). Miniaturized Tapered Photonic Crystal Fiber Mach–Zehnder Interferometer for Enhanced Refractive Index Sensing. IEEE Sens. J..

[33] Li L., Xia L., Xie Z., Liu D. (2012). All-fiber Mach–Zehnder interferometers for sensing applications. Opt. Express.

[34] Quintero S.M.M., Valente L.C.G., de Paula Gomes M.S., da Silva H.G., de Souza B.C., Morikawa S.R.K. (2018). All-fiber CO_2_ sensor using hollow core PCF operating in the 2 µm region. Sensors.

